# Inhibition of Cell Division Induced by External Guide Sequences (EGS Technology) Targeting *ftsZ*

**DOI:** 10.1371/journal.pone.0047690

**Published:** 2012-10-23

**Authors:** Carol Davies Sala, Alfonso J. C. Soler-Bistué, Leeann Korprapun, Angeles Zorreguieta, Marcelo E. Tolmasky

**Affiliations:** 1 Fundación Instituto Leloir-Instituto de Investigaciones Bioquímicas Buenos Aires, Consejo Nacional de Investigaciones Científicas y Técnicas – Facultad de Ciencias Exactas y Naturales, Universidad de Buenos Aires, Buenos Aires, Argentina; 2 Center for Applied Biotechnology Studies, Department of Biological Science, College of Natural Sciences and Mathematics, California State University Fullerton, Fullerton, California, United States of America; University of Arizona, United States of America

## Abstract

EGS (external guide sequence) technology is a promising approach to designing new antibiotics. EGSs are short antisense oligoribonucleotides that induce RNase P-mediated cleavage of a target RNA by forming a precursor tRNA-like complex. The *ftsZ* mRNA secondary structure was modeled and EGSs complementary to two regions with high probability of being suitable targets were designed. *In vitro* reactions showed that EGSs targeting these regions bound *ftsZ* mRNA and elicited RNase P-mediated cleavage of *ftsZ* mRNA. A recombinant plasmid, pEGSb1, coding for an EGS that targets region “b” under the control of the T7 promoter was generated. Upon introduction of this plasmid into *Escherichia coli* BL21(DE3)(pLysS) the transformant strain formed filaments when expression of the EGS was induced. Concomitantly, *E. coli* harboring pEGSb1 showed a modest but significant inhibition of growth when synthesis of the EGSb1 was induced. Our results indicate that EGS technology could be a viable strategy to generate new antimicrobials targeting *fts*Z.

## Introduction

Bacterial cell division is a complex process that occurs following the replication and segregation of chromosomal DNA to the two halves of the growing cell. In the case of Gram negative bacteria, the division process requires at least 14 cytoplasmic, membrane and periplasmic proteins, of which 10 are essential [1]–[5]. These proteins form a structure known as the divisome, a ring-like cell division complex located at midcell that constricts during division and disappears when the cells separate [1]–[5]. Assembly of the divisome starts with the formation of the proto-ring, a complex formed by FtsZ, FtsA, and ZipA, and continues with the assembly of other proteins and protein complexes [6], [7]. The temporal events of the assembly of the divisome have recently been established with high resolution in *Caulobacter crescentus* [8]. The most conserved of all known bacterial cell division genes is the proto-ring protein FtsZ, which functions as scaffold for the divisome and generates the constrictive force to initiate division of the cell [6], [9], [10]. These properties, together with the fact that it does not share significant sequence similarity to the eukaryotic cytoskeletal protein tubulin made FtsZ an ideal choice as target for drug discovery [11]. Numerous reports have been published proposing novel cell division inhibitors that act by blocking FtsZ and hold high therapeutic potential but none of them have been fully developed and released to the market to date [11]–[18].

A promising approach to design new antimicrobial agents is based on the properties of the ribozyme RNase P, a ribonucleoprotein composed of an RNA component (M1) that is the catalytic subunit and a cofactor protein (C5). RNase P plays an essential role in the cell by directing maturation of RNA species by precise cleavage of molecules such as precursor t-RNAs or some polycistronic mRNAs [19]. The finding that RNase P can be induced to digest target RNA molecules that are not natural substrates by addition of an appropriate complementary oligoribonucleotide, known as “external guide sequence” (EGS), led to development of what is known as EGS technology [20]. For efficient degradation of the target RNA, the EGS must form a duplex that results in the appropriate stem-like structure required to serve as substrate of RNase P [21]. EGS technology has been used to inhibit expression of several genes coding for essential housekeeping functions, virulence factors, and antibiotic resistance enzymes [20], [22]–[29]. In the first demonstration that EGSs could be used to turn off bacterial genes, oligoribonucleotides encoded by plasmids elicited about 50% reduction in expression levels of β-galactosidase and alkaline phosphatase [30]. Later on, phenotypic conversion to susceptibility was shown targeting resistance genes such as *cat*, *bla*, and *aac(6′)-Ib* in *E. coli* [26]–[28]. Examples of essential and virulence genes whose expression has been successfully reduced by using EGS technology are the *E. coli gyrA* and *rnpA* genes [25], the *Salmonella* Typhimurium *invB* and *invC* genes, which resulted in diminished secretion of proteins that are exported using the type III secretion system and impairment of the ability to invade host cells [24], the *Francisella tularensis mglB* gene [22], and the *Yersinia pestis yscN*, and *yscS* genes [23]. In the present study we designed an EGS complementary to the *Escherichia coli ftsZ* mRNA that interferes with cell division.

## Materials and Methods

### Bacterial Strains and Plasmids

*E. coli* BL21(DE3)(pLysS) F^−^ *dcm ompT hsdS*(r_B_^−^m_B_^−^) *gal* λ(DE3) pLysS [31] was used as host for the recombinant plasmids coding for the EGSs. *E. coli* DH5α was used for regular cloning experiments. Bacterial cultures were carried out in Lennox Luria (L) broth [32]. Recombinant plasmids pEGSb1 and pEGSb1S were generated by inserting a DNA fragment including the T7 promoter (GCGAAATTAATACGACTCACTATAGGG) followed by the EGS sequence (EGSb1 or EGSb1S) (Table 1), the consensus ACCA sequence, a hammerhead core [30], and a T7 terminator sequence (TAGCATAACCCCTTGGGGCCTCTAAACGGGTCTTGAGGGGTTTTTTG) into the *Xba*I and *Bam*HI sites of the cloning vehicle pUC57 (GenBank/EMBL accession no. Y14837).

**Table 1:** EGS sequences.

EGS name	Sequence	*ftsZ* region targeted
EGSa1	AUAGUGAUCAGAGACCA	697–709
EGSa2	GAUAGUGAUCAGAGAACCA	696–710
EGSb1	CCGUUUCGAACUCACCA	1041–1053
EGSb1S	GAGUUCGAAACGGACCA	Not applicable (control)
EGSAP	AGGCATCTATACCACCA	Not applicable (control)

EGSAP targets the *phoA* gene. EGSb1S is the sequence complementary to EGSb1.

### General Procedures

Plasmid DNA preparations were carried out using the Wizard® Plus SV Minipreps DNA Purification System (Promega). Polymerase chain reactions (PCR) were carried out using the HotStar Taq master mix kit (QIAGEN). All endonuclease restriction and ligase treatments were performed according to the supplier’s recommendations (New England Biolabs). *In vitro* synthesis of RNA molecules to generate the *ftsZ* mRNA was done using a MEGAshortscript high-yield transcription T7 kit (Life Technologies) according to the protocols provided by the supplier. RNase P was prepared by mixing *in vitro* synthesized M1 RNA and purified C5 as described previously [28]. Denaturing polyacrylamide gel electrophoresis (PAGE) was performed as described previously [33] on 6% polyacrylamide 16∶1 (acrylamide-bis-acrylamide) gels using a glycerol-tolerant gel (GTG) buffer containing, 7 M urea, 89 mM Tris, 29 mM taurine, and 0.5 mM EDTA (USB Corp.). Electrophoretic mobility shift assays were carried out using 6% polyacrylamide native (non-denaturing) gels prepared with TBE buffer (89 mM Tris Base, 89 mM boric acid, 2 mM EDTA pH 8.0) [33]. Radioactivity was visualized using a STORM 840 PhosphorImager (Molecular Dynamics). M1 RNA, *ftsZ* mRNA, and C5 protein were synthesized or purified as described before [28]. Oligoribonucleotides were obtained from a commercial source (IDT Technologies). The computer-predicted secondary structure of the *ftsZ* mRNA was generated by using the m-fold program (version 3.1) [34], [35].

### *In vitro* RNase P Assays

The nucleotide sequences of the EGSs are shown in Table 1. EGSs were tested to determine their ability to elicit RNase P-mediated cleavage by preincubating 5′-end-radiolabeled *ftsZ* mRNA (0.25 pmol) and the EGS (50 pmol) at 25°C for 2 h in a volume of 3 µl before adding this mixture to a solution containing 2.5 pmol of M1 RNA, 70 pmol of C5 protein, 20 mM HEPES-KOH (pH 8.0), 400 mM ammonium acetate, 10 mM magnesium acetate, and 5% glycerol that had been preincubated at 37°C for 15 min in a final volume of 7 µl [36]. After combining both solutions the mix was incubated at 37°C for 90 minutes, the reaction was stopped by the addition of 1 volume of gel loading buffer, and analyzed by 6% denaturing GTG-PAGE as described before [27].

### Binding Assays

Gel shift assays were carried out mixing 5′-end-labeled oligoribonucleotides (1 µM) with (1, 10, 100, or 500 ng) or without *ftsZ* mRNA in binding buffer (150 mM NaCl, 10 mM Tris-HCl pH 8.0, 1 mM EDTA) in a final volume of 10 µl. The mix was incubated for 1 h at 25°C and after addition of 10 µl of 10% glycerol and 4 µl of gel loading buffer the samples were analyzed on native TBE-PAGE (acrylamide-bisacrylamide [19∶1]).

### *In vivo* Activities of EGSs

Each *E. coli* BL21(DE3)(pLysS) harboring a recombinant plasmid strain was cultured in L broth containing 100 µg/ml ampicillin and 20 µg/ml chloramphenicol at 37°C until the optical density at 600 nm (OD_600_) reached 0.15–0.2. At this moment expression of the EGS was induced by addition of 100 µM or 1 mM isopropyl-β-D-thiogalactopyranoside (IPTG) and the culture was incubated for 60 or 90 minutes before the cells were examined by microscopy or were plated to determine colony forming units (CFU). For regular optical microscopy examination, the cells were spread on a slide, fixed by heat and stained with violet crystal (magnification 1000X). For laser scanning confocal microscopy bacteria were washed and resuspended in 20 µl saline buffer; then FM 5–95 was added to a final concentration of 6.5 µg/ml and placed on ice for 5 minutes. Cells were then poured on 2% agar, covered, and examined (magnification 400X). For CFU determination, cells in culture were serially diluted, spread on LB plates and colonies were counted after overnight incubation at 37 °C. The experiments were repeated 3 times (twice by sextuplicate and once by quadruplicate) and the results are expressed as mean log_10_(CFU/ml) ± SD. Statistical significance was analyzed by an unpaired two-tailed t-test. P<0.01 was considered statistically significant.

## Results

### *In vitro* EGS/*ftsZ* mRNA Binding and RNase P-mediated Cleavage of *ftsZ* mRNA

**Figure 1:**
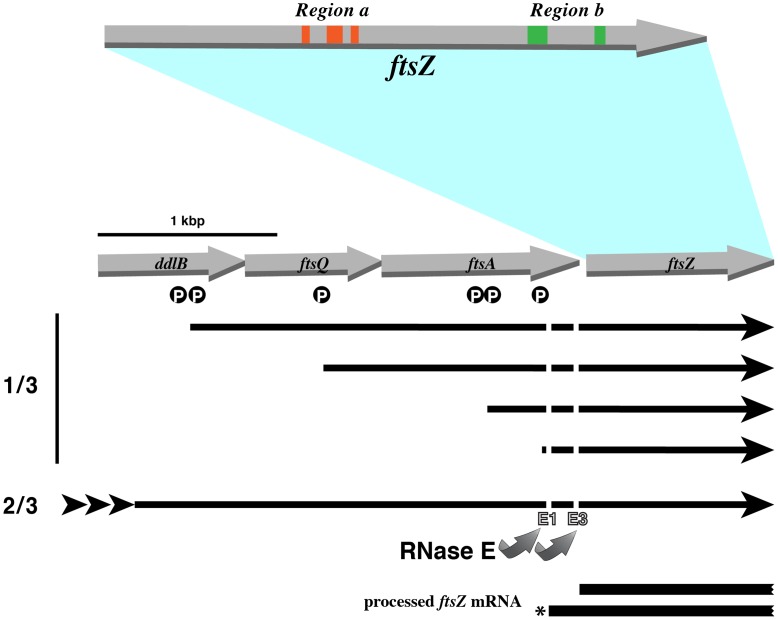
Genetic organization of the relevant region of the *dcw* cluster. The grey arrows represent genes, the black arrows represent mRNA molecules, and the white P inside a black circle represent locations of the promoters within *ddlB, ftsQ*, and *ftsA*. The long mRNAs originated at promoters upstream of *ddlB* are represented by a black arrow with arrowheads at the beginning. The diagram shows that about 1/3 of the transcription of *ftsZ* originates at the promoters located within *ddlB, ftsQ*, and *ftsA* and about 2/3 of the transcription is initiated at the far upstream promoters [38]–[40]. The E1 and E3 RNase E cleavage sites are indicated by curved arrows. The asterisk shows the RNA molecule utilized in this work to identify EGSs that elicit RNase P-mediated cleavage of the *ftsZ* mRNA [40]. The regions (“a” and “b”) used to design EGSs are shown in the same colors used in Figure 2.

The *E. coli ftsZ* gene is located towards the distal end of the *dcw* cluster, a group of 16 genes involved in cell division and cell wall synthesis [37]. This cluster possesses a complex genetic organization and several promoters within the immediately upstream *ddlB*, *ftsQ* and *ftsA* genes as well as distant upstream promoters contribute to *ftsZ* expression (Figure 1) [38]. About one third of the *ftsZ* transcripts are originated at the promoters located closely upstream of this gene (Figure 1) [38]–[40]. Regardless of the promoter they are transcribed from, the bulk of the *ftsZ* encoding mRNAs are precisely processed by RNAse E at specific sites close to the location of translation initiation. Two species, originated at digestion sites E1 and E3, are required for appropriate cell division (Figure 1) [38], [40]–[44]. Therefore, to identify EGSs that elicit RNase P-mediated cleavage of the *ftsZ* mRNA we synthesized a molecule that includes 221 nucleotides of the 5′-UTR for our analysis.

**Figure 2:**
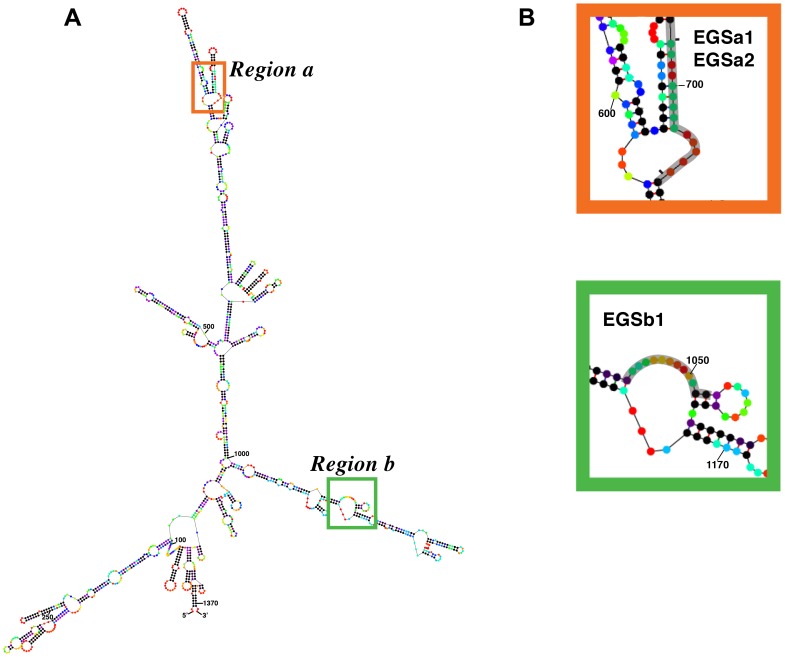
Secondary structure of *ftsZ* mRNA and regions targeted by EGSs. A. Secondary structure of the *ftsZ* mRNA (nucleotides 105083–106456, accession number NC_000913.2) generated with m-fold software [34]. B. Zoom in the two regions selected as targets. Colors of the dots indicate the probability that they exist as single stranded. In decreasing order: red, orange, yellow, green, cyan, blue, violet and black. The sequences targeted by the EGSs are shown shadowed. EGSa2 includes an extra nucleotide at each end with respect to EGSa1, this is indicated by two short lines.

**Figure 3:**
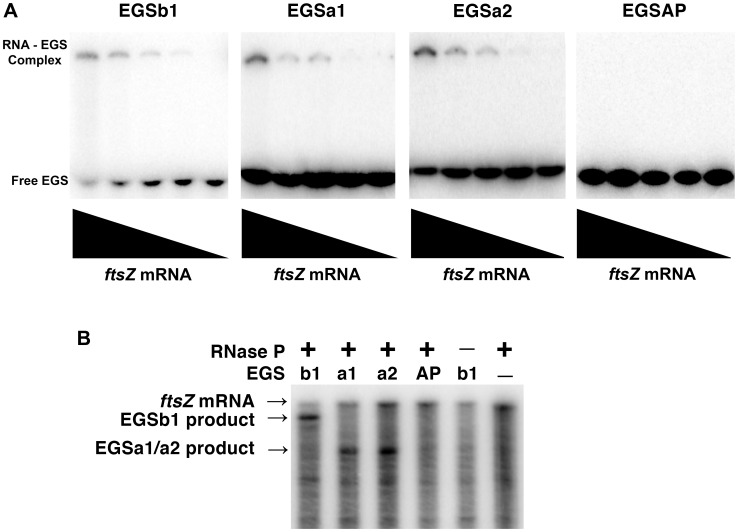
Analysis of the activity of EGSs. A. Binding of EGSs to *ftsZ* mRNA. The oligoribonuclotides were 5′-end labeled, mixed with different amounts of *ftsZ* mRNA (from left to right 0, 1, 10, 100, or 500 ng) and analyzed by electrophoresis in 6% native polyacrylamide gel. B. RNase P-mediated cleavage of ^32^P-labeled *ftsZ* mRNA. The RNase P components, M1 RNA and C5 protein were preincubated at 37°C for 15 min, and a mix containing the radiolabeled *ftsZ* mRNA and the indicated EGS was preincubated 25°C for 2 h. After preincubation both solutions were combined, incubated at 37°C for 90 minutes, and analyzed on 6% denaturing PAGE. The location and size of the expected products of cleavage are shown to the left.

We first identified regions within the *ftsZ* mRNA that may be accessible for interaction with complementary oligoribonucleotides by m-fold analysis (Figure 2A and Figure S1, the regions are also indicated in Figure 1). We selected two *ftsZ* mRNA regions containing numerous nucleotides that are predicted to have high probability of existing as single stranded and their structures resemble those we found in the past to be good candidates as EGSs (Figure 2B) (27,28). We then designed EGSs to target these regions; their sequences as well as the sequences of control EGSs are shown in Table 1. Electrophoretic mobility shift assays using the EGSs and *ftsZ* mRNA showed that all three EGSs targeting regions “a” or “b” bound the *ftsZ* mRNA (Figure 3A). The efficiency of these EGSs to elicit RNase P-mediated cleavage of *ftsZ* mRNA was determined *in vitro* incubating the oligoribonucleotides and labeled mRNA with the components of RNase P, M1 RNA and C5 protein. Figure 3B shows that both regions are efficient targets for RNase P in the presence of EGSa1, EGSa2, or EGSb1. All three EGSs induced significant cleavage of the *ftsZ* mRNA at the expected locations (Figure 3B). In the case of region “a” we tested two different sizes of the antisense portion of the EGSs: EGSa1, 13 nucleotides, and EGSa2, 15 nucleotides. No significant differences were observed in RNase P cleavage eliciting activity (Figure 3B). As expected, negative controls consisting of incubation in the absence of an EGS or RNase P, or in the presence of an EGS targeting the *phoA* gene showed no *ftsZ* mRNA-degradation activity (Figure 3B).

### EGS-induced Filamentation

**Figure 4:**
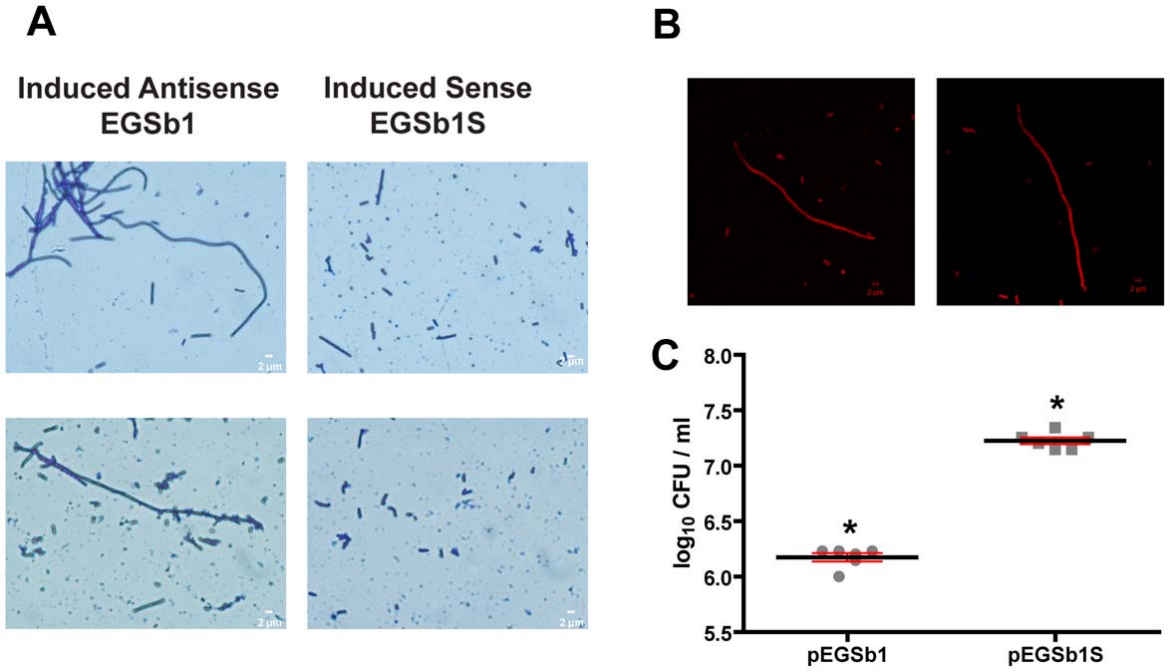
Effect of EGSb1 on cell division. A. The inducer IPTG (1 mM) was added to cultures of *E. coli* BL21(DE3)(pLysS) harboring the recombinant plasmids pEGSb1 or pEGSb1S when their OD_600_ reached 0.2. The cultures were then incubated for 60 more minutes and cells were examined by microscopy. B. Cells were stained with FM5-95 as described in Materials and Methods and examined by laser scanning confocal microscopy. C. Effect of the expression of EGSb1 or EGSb1S on cell survival. Results are expressed as log_10_ of mean CFU/ml ± SD. Similar results were observed in 3 independent assays. *: p<0.01.

We selected EGSb1 to test its ability to interfere with cell division. We generated a recombinant plasmid with an insert that includes a T7 promoter followed by the EGSb1 coding region, the ACCA sequence, which enhances RNase P-substrate recognition; and a sequence required to generate a hammerhead ribozyme to generate the correct 3′ terminus of the EGSb1 by *cis* cleavage as described before [26]. *E. coli* BL21(DE3)(pLysS) was transformed with pEGSb1 or the control plasmid pEGSb1S, which codes for EGSb1S, whose sequence is complementary to EGSb1. IPTG was added to cultures in exponential growth phase followed by incubation for 60 or 90 minutes and cell analysis by microscopy. We consistently observed filamentation in the cultures of cells harboring pEGSb1 (Figure 4A). However, the proportion of filaments varied in different cultures. Although we still do not know the reason behind these differences, our results show a clear effect mediated by EGSb1 (see Figure 4A). Staining the filaments with FM5-95 suggested that the filaments are individual cells as no evidence of septa was detected (Figure 4B). Further experiments will permit us to confirm if there is complete or partial lack of septa. The CFU/ml were determined for cultures of both *E. coli* BL21(DE3)(pLysS) harboring pEGSb1 or pEGSb1S. Figure 4C shows that there was a significant reduction in the CFU/ml in the cultures of cells producing EGSb1 confirming that this EGS has a detrimental effect, most probably due to the observed inhibition of cell division.

## Discussion

Antimicrobial resistance has been identified as one of the greatest threats to human health [45]. The quick increase in resistant strains observed among a number of bacterial pathogens together with the low number of candidate compounds existing in the pipeline warrant the need to look for alternatives to design new antibiotics [45], [46]. FtsZ has been proposed and used numerous times as target for developing new antimicrobials [12], [13], [15]–[17]. However, to our best knowledge EGS inhibition of expression had not been tried on this cell division gene. EGSs were designed to target two regions within the *ftsZ* mRNA and tested to determine their mRNA binding properties, as well as their efficiency to induce RNase P-mediated degradation *in vitro*. The EGSs that target regions “a” and “b” showed significant binding capabilities and ability to elicit RNase P-mediated degradation of the *ftsZ* mRNA. EGSb1 showed activity *in vivo* as the presence of pEGSb1 within *E. coli* resulted in filamentation upon addition of IPTG to the culture, a phenotype that was observed when other strategies were used to target FtsZ [12], [13], [47], [48]. As expected, induction of expression of EGSb1 also resulted in growth impairment. Although EGS technology is still at an early stage, development of appropriate EGSs that can inhibit expression of resistance genes [26]–[28] or act themselves as antibiotics [23]–[25], [29] could be a way to keep ahead of the race between availability of antibiotics and development of multiresistance. Here we show that EGSs could be developed to interfere with cell division. However, since recombinant clones coding for EGSs do not represent a realistic recourse for their practical application we are presently developing nuclease resistance alternatives with the EGSb1 sequence that will induce impairment of cell division through interference with proper FtsZ expression. We have recently shown that hybrid oligomers consisting on locked nucleic acids (LNA) and DNA residues (LNA/DNA) have activities comparable to those shown by isosequential oligoribonucleotides [27]. Another hurdle for reducing to practice the utilization of LNA/DNA EGSs as antibacterial treatment is the lack of a viable methodology for their uptake by bacterial cells. Some encouraging results have recently been reported on gymnotic delivery of LNA containing oligomers and cell internalization of oligonucleotides and analogs using strategies such as liposome encapsulation or attachment of cell-permeabilizing peptides to peptide nucleic acids or phosphorodiamidate morpholino oligomers [29], [49]–[52]. Unfortunately, attempts to conjugate cell-permeabilizing peptides to LNA/DNA oligomers have so far been unsuccessful, most probably due to the negatively charged nature of these compounds. However, other solutions such as gymnotic delivery [52]–[54] in appropriate conditions or co-administration with lipopeptide transfection agents remain to be explored [55]–[60].

In conclusion, the results shown in the present study indicate that, after overcoming existing stumbling blocks, the development of RNase P-mediated *ftsZ* mRNA degradation could contribute to the need for developing new antibiotics.

## Supporting Information

Figure S1 **ss count plot.** The ss count plot is the propensity of a base to be single stranded as determined by the number of times it is single stranded in a group of predicted foldings, in this case 33, (http://mfold.rna.albany.edu/?q=mfold/documentation). To simplify the interpretation, the length of the untranslated region (black bar) and the coding region of ftsZ (arrow) has been superimposed to the plot.
(ZIP)
