# Supplementary figures and images for: Inhibition of Cell Division Induced by External Guide Sequences (EGS Technology) Targeting ftsZ
Source: PLoS One. 2012 Oct 23;7(10):e47690. doi: 10.1371/journal.pone.0047690 (PMC3479136; doi:10.1371/journal.pone.0047690)

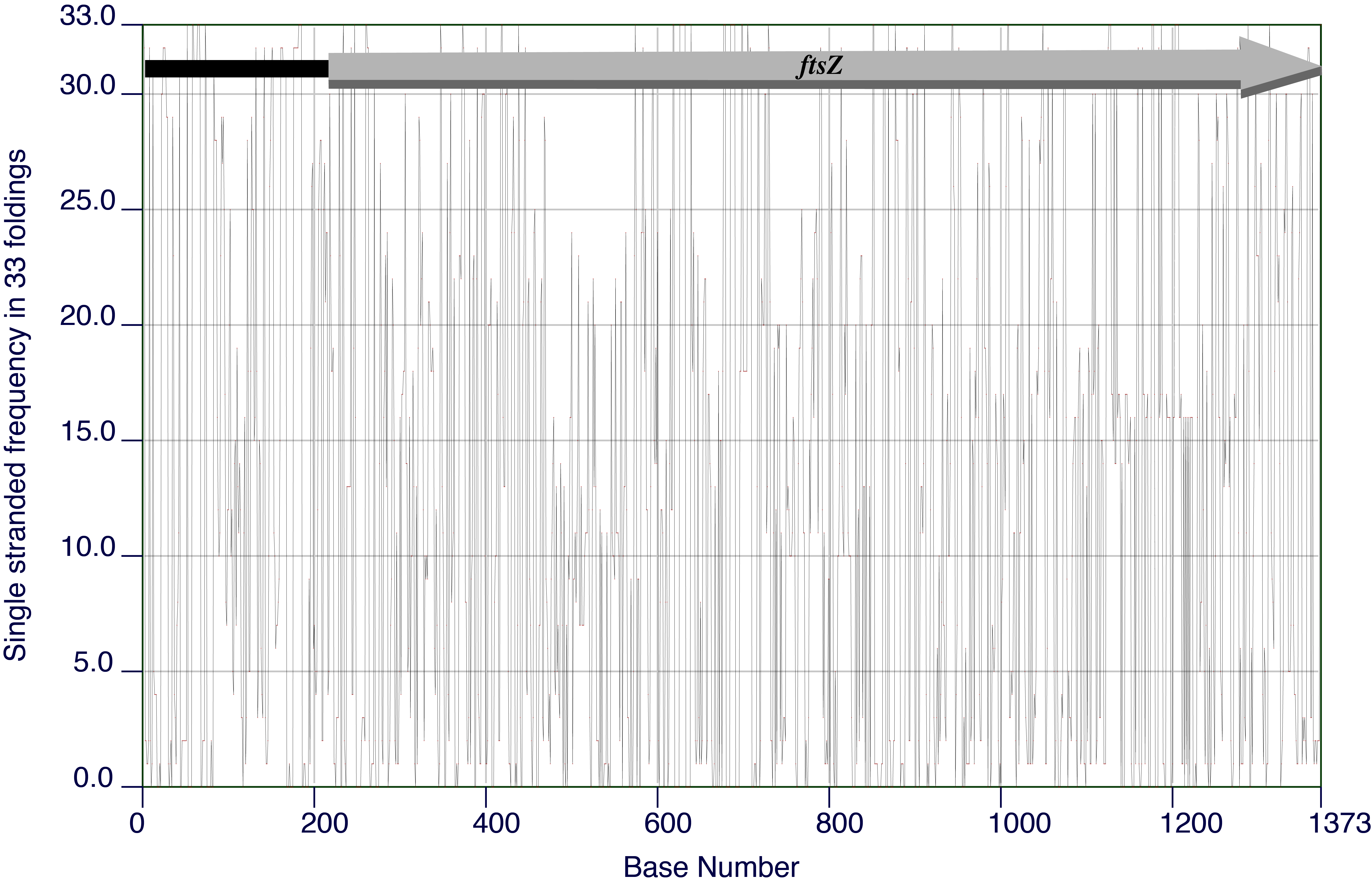

Supplement: Figure S1 — ss count plot. The ss count plot is the propensity of a base to be single stranded as determined by the number of times it is single stranded in a group of predicted foldings, in this case 33, (http://mfold.rna.albany.edu/?q=mfold/documentation). To simplify the interpretation, the length of the untranslated region (black bar) and the coding region of ftsZ (arrow) has been superimposed to the plot. (ZIP) [file pone.0047690.s001.zip]
